# Proactive Caching in D2D Assisted Multitier Cellular Network

**DOI:** 10.3390/s22145078

**Published:** 2022-07-06

**Authors:** Fawad Ahmad, Ayaz Ahmad, Irshad Hussain, Ghulam Muhammad, Zahoor Uddin, Salman A. AlQahtani

**Affiliations:** 1 Department of Electrical and Computer Engineering, COMSATS University Islamabad, Wah Campus, Wah 47040, Pakistan; fawadkhan@uetpeshawar.edu.pk (F.A.); ayaz.ahmad@ciitwah.edu.pk (A.A.); zahooruddin@ciitwah.edu.pk (Z.U.); 2 Faculty of Electrical and Computer Engineering, University of Engineering and Technology Peshawar, Peshawar 25000, Pakistan; 3 Research Chair of New Emerging Technologies and 5G Networks and Beyond, Computer Engineering Department, College of Computer and Information Sciences, King Saud University, Riyadh 11543, Saudi Arabia; salmanq@ksu.edu.sa; 4 Department of Computer Engineering, College of Computer and Information Sciences, King Saud University, Riyadh 11543, Saudi Arabia

**Keywords:** cache-enabled D2D transmitter, multitier cellular networks, content delivery, content caching, 5G

## Abstract

Cache-enabled networks suffer hugely from the challenge of content caching and content delivery. In this regard, cache-enabled device-to-device (D2D) assisted multitier cellular networks are expected to relieve the network data pressure and effectively solve the problem of content placement and content delivery. Consequently, the user can have a better opportunity to get their favored contents from nearby cache-enabled transmitters (CETs) through reliable and good-quality links; however, as expected, designing an effective caching policy is a challenging task due to the limited cache memory of CETs and uncertainty in user preferences. In this article, we introduce a joint content placement and content delivery technique for D2D assisted multitier cellular networks (D2DMCN). A support vector machine (SVM) is employed to predict the content popularity to determine which content is to be cached and where it is to be cached, thereby increasing the overall cache hit ratio (CHR). The content request is satisfied either by the neighboring node through the D2D link or by the cache-enabled base stations (BSs) of the multitier cellular networks (MCNs). Similarly, to solve the problem of optimal content delivery, the Hungarian algorithm is employed aiming to improve the quality of satisfaction. The simulation results indicate that the proposed content placement strategy effectively optimizes the overall cache hit ratio of the system. Similarly, an effective content delivery approach reduces the request content delivery delay and power consumption.

## 1. Introduction

In the recent era, although various technologies have been developed in the field of wireless communication, device-to-device (D2D) communication can be distinguished. This technology has received extensive attraction due to the fact that it can communicate directly within a certain range of distance without the assistance of an intermediate node, thus reducing the huge cost of back haul and transmission power [1,2]. In addition to this, the emergence of edge caching technology enables users to get their favored contents from their neighboring cache-enabled nodes, thus reducing latency and enhancing user experience [3] and satisfaction ratio [4]. Similarly, the introduction of multitier networks brings the network closer to the user, thus enhancing the link quality [5]. Therefore, the combination of these three techniques allows embarking on a new research arena for researchers in data traffic offloading in wireless communication. Consequently, the user can get their favored contents with little delay through good links. Highly favored contents can be cached in D2D transmitters and in the BSs of the multitier cellular networks in advance. If the requested contents are cached in neighboring D2D transmitters, then a request is served; otherwise, the request is sent to the cache-enabled multi-tier cellular networks. Every new technology has certain issues. The same is the case with proactive caching. Here, one of the big issues is content placement. The content placement determines the amount of data unloaded from the cache-enabled nodes. Hence, the cache hit ratio plays a vital role [6,7]. In this regard, the popularity of the contents should be known in advance for optimal cache placement [8,9,10,11]. Hence, if the contents are cached properly in the cache-limited memory of caching nodes, then users can download their requested contents from neighboring nodes within a short distance, thus achieving a high data rate. Similarly, the same content may be requested by different users simultaneously or the request of a user may be satisfied by multiple transmitters. Thus, content delivery needs a proper mechanism. In the nest section of this research article, we introduce some results and limitations prior to our work.

### 1.1. Motivation and Related Work

The existing literature includes huge data related to various proactive caching and content delivery techniques. The authors in [12,13] introduced probabilistic caching schemes by optimizing the D2D link and reliability of transmission. Similarly, the author in [14] used the Indian buffet process (IBP) to increase the caching performance by predicting the content requests. System performance was also improved by estimating the future contents requests using logistic regression models [15,16]. The author in [17] also designed new caching techniques considering user preferences. The problem of data sparsity in [18] was solved using collaborative filtering (CF) and the latent factor model. For better prediction, a deep learning algorithm was also used, which fairly and accurately predicted content popularity [19,20]. Stochastic geometry was used to address the problem of contents in terms of CHR, the density of served requests, and power consumption. In [21], the Poisson point process (PPP) solved the problem of optimal geographical content placement. Golrezaei et al. [22] introduced a caching technique for each base station (BS) to reduce the downloading delay. The survey in [23] discussed caching in two-tier small networks. In [24], the performance analysis of content caching in three-tier heterogeneous networks was discussed. However, few studies have utilized the concept of multitier caching. For instance, in [25], the authors discussed the performance analysis of content caching in three-tier heterogeneous networks while keeping content size identical. In [26], the authors suggested a caching mechanism in two-tier networks. However, the authors in [25,26] did not discuss the different caching strategies with practical considerations for limited cache. Similarly, the authors in [27,28,29] suggested different content delivery schemes from BSs to users. However, these studies were only limited to single-tier caching. On other hand, few studies have been conducted on small base station D2D (SBS-D2D) caching, again limited to single-tier only. For example, in [30], for SBS-D2D caching, an optimal cooperative-based caching policy was discussed. The authors in [31] suggested an intelligent caching technique based on Z-Pf request distribution in cache-enabled D2D networks. Similarly, the authors in [32] proposed user mobility-based content caching and solved energy and delay minimization using reinforcement learning, albeit restricted to D2D networks. In contrast to all existing studies, we combined cache-enabled D2D networks and cache-enabled multitier cellular networks to solve the problems of content caching and content delivery using SVM and the Hungarian algorithm, respectively. SVM is one of the best learners and is used in wireless communication in different scenarios [33,34,35]. It uses the radial basis function (RBF) as a kernel for transformation. The RBF can better map feature vectors into a nonlinear space as compared to other learners [36,37,38]. On the other hand, the Hungarian algorithm is also already utilized to solve various assignment issues such as resource allocation in wireless communication systems [39,40,41].

### 1.2. Contributions and Outcomes

The existing work mainly focused on caching favored contents in cache-enabled D2D networks. This article not only exclusively encompasses the proactive caching issues but also solves the delay and power consumption minimization problems in D2D assisted cache-enabled multitier cellular networks (D2DMCNs).

Two different algorithms are employed, one for content caching and the second one for power and delay minimization. The support vector algorithm predicts the popularity of the contents, and the Hungarian algorithm optimizes the delay and power consumption on the basis of a reward function. The content popularity prediction is based on content attributes such as the number of likes, numbers of dislikes, shares, and first-day view counts. Cache placement is carried out in the D2DMCN after predicting first-day view counts.

Although limitations exist, the addition of D2D technology to MCN greatly increases the satisfaction ratio of the users. As there is no free lunch, an agreement between D2D users and the cellular network operator is necessary. Therefore, as an incentive, extra free MBs are allocated for D2D transmitters, as their memories and battery power are used for content caching and content delivery, although incentive-based mechanisms are not discussed in this paper. This allocation of extra MBs is bounded to the memory and battery power of the D2D transmitter volunteered for proactive caching.

The major contributions of this research paper are described below.

#### 1.2.1. Novel Caching Scheme

In this research work, a joint content caching and content delivering scheme is developed to solve the cache hit problems, content delay, and power requirements in D2DMCN. This content caching policy considers different contents attributes for their popularity. The most popular contents are placed in incentivized D2D transmitters (ID2DTs) and MCN. A picocell, microcell, and macrocell constitute an MCN.

#### 1.2.2. Implementation of Content Placement Techniques

In this research, we use the SVM-based learning algorithm to predict content popularity. Then, on the basis of SVM’s predictions, content placement is carried out in ID2DTs and MCN. Hence, the cache hit ratio is maximized, and the delay and power consumption are minimized.

#### 1.2.3. Content Delivery Decision Making

The content delivery problem in a cache-enabled D2DMCN is formulated as an assignment problem, where the distances of the D2D receivers (D2DRs) from ID2DTs and BSs of the MCN, channel gain, and transmission power are considered as inputs. A dynamic content delivery link is established between D2DRs and ID2DTs or between D2DRs and BSs of the MCN with the goal of minimizing the content delivery delay and power consumption, thereby solving the problem of content delivery using the Hungarian algorithm.

#### 1.2.4. Maximization of Objective Function

A reward function is constructed, consisting of power consumption and content delay. Contents may be delivered from ID2DTs or BSs of the MCN depending on their reward function.

A simulation is conducted from the perspective of the cache-enabled ID2DTs, cache-enabled MCN, and D2D receiver. The weight coefficients of the reward function are adjusted so that the content delivery problem is optimized. Hence, the D2DRs receive contents from neighboring ID2DTs or the MCN.

Clusters based on similarities of ID2DTs are formed using K-means clustering. The ID2DTs are selected, and clusters are further squeezed into those D2DRs which are in the threshold distance range of the selected ID2DTs.

## 2. System Model 

**Figure 1:**
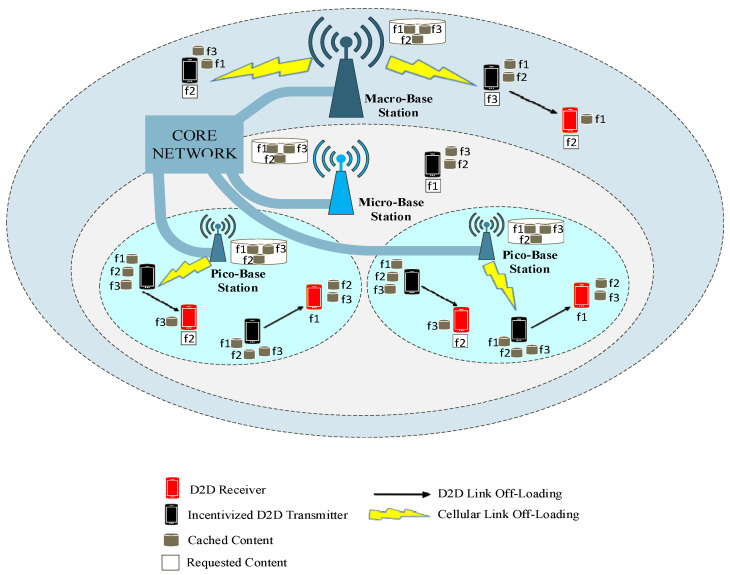
Cache-enabled D2D assisted multitier cellular network.

As shown in Figure 1, consider a cache-enabled D2DMCN consisting of cache-limited pico BSs, micro BSs, and macro BSs. The ID2DTs are also employed to serve the neighboring D2DRs. Popular contents are cached in ID2DTs and in the cache-enabled BSs of MCN. The contents are cached from a content library set ℱ={1,2,…,f,….F}. The ID2Ds set is K={1,2,…….,i,……k} and D2DRs set is ℛ={1,2,3,……..,j,……R}. ID2DTs can store $f_{u}$ files while pico BSs, micro BSs, and macro BSs can store up to $f_{p},f_{m},$ and $f_{M}$ files, respectively. ID2DTs have limited cache memory $s_{D2D}$ while $S_{p},\;S_{m},$ and $S_{M}$ are the cache memories of pico BSs, micro BSs, and macro BSs, respectively.

**Figure 2:**
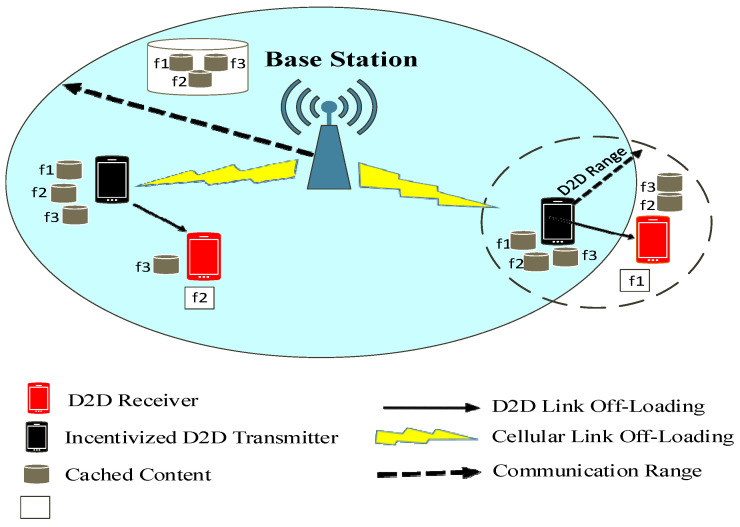
Formation of squeezed cluster in proximity of ID2DTs.

The delay and power consumption in cellular communications are huge as compared to D2D communication. Consequently, the user satisfaction rate is high as they are served by ID2DTs lying in the neighborhood. However, this can be accomplished only when they are in the threshold distance range $d_{max}\;$ of the ID2DTs. Otherwise, the signal quality will be very bad. If a D2DR finds its requested contents in the neighboring ID2DTs, it establishes a D2D link with them; otherwise, it sends its request to the cache-enabled MCN to offload the contents via cellular link. Each file size is considered to be the same; mostly, at each time slot, the D2DR can only request one file, and ID2DTs can send one file. The D2D users with the same content requirements form a virtual cluster. This cluster is further squeezed, and only those D2D users which are in the threshold range of the ID2DTs occupy the cluster. Figure 2 shows the formation of a squeezed cluster in proximity of the ID2DTs. The contents can be cached in pico BSs, micro BSs, and macro BSs depending on the behavior of their users. The contents are placed in ID2DTs for some allocated time and then they are replaced by new contents. The time allocation for contents to be present in the cache memory of the devices depends on the variation in the popularity of the favored contents.

## 3. Problem Formulation

In cache-enabled cellular networks, prime issues are what to cache and where to cache, as well as how to deliver and from where to deliver. In addition to this, efficient prediction of popularity of contents is also necessary for maximization of the cache hit ratio and minimization of the delay and power consumption. All of these problems directly affect the efficiency of cache-enabled cellular communication systems. In order to formulate our problems, we have to define certain system parameters. Table 1 shows the system-related parameters and their descriptions.

Any D2DR positioned within the proximity of the ID2DTs is considered to be in the squeezed cluster (SC), i.e., if $\lambda_{i,j}=1$, then D2DR j exists within the proximity of ID2DT i. Otherwise, $\lambda_{i,j}=0$.

Similarly, D2DRs are connected in one of the following modes: (i) D2D mode, (ii) pico mode, (iii) micro mode, or (iv) macro mode.

If ${\left(d_{i,j}\right)}^{-\eta_{d}}\geq {\left(d_{p,j}\right)}^{-\eta_{p}}$, then $Z_{i,j}=\left\{\begin{array}{l} 1,\;D2DR\;is\;\mathrm{connected}\;\mathrm{in}\;D2D\;\mathrm{mode}\\ 0,\;\mathrm{otherwise} \end{array}\right\}$.

If ${\left(d_{p,j}\right)}^{-\eta_{p}}\geq {\left(d_{m,j}\right)}^{-\eta_{m}}$, then $Z_{P,j}=\left\{\begin{array}{l} 1,\;D2\mathrm{DR}\;\mathrm{is}\;\mathrm{connected}\;\mathrm{in}\;\mathrm{pico}\;\mathrm{mode}\\ 0,\;\mathrm{otherwise} \end{array}\right\}$.

If ${\left(d_{m,j}\right)}^{-\eta_{m}}\geq {\left(d_{M,j}\right)}^{-\eta_{M}}$, then $Z_{m,j}=\left\{\begin{array}{l} 1,\;D2\mathrm{DR}\;\mathrm{is}\;\mathrm{connected}\;\mathrm{in}\;\mathrm{micro}\;\mathrm{mode}\\ 0,\;\mathrm{otherwise} \end{array}\right\}$.

Otherwise, $Z_{M,j}=\left\{\begin{array}{l} 1,\;D2\mathrm{DR}\;\mathrm{is}\;\mathrm{connected}\;\mathrm{to}\;\mathrm{macro}\;\mathrm{mode}\\ 0,\;D2\mathrm{DR}\;\mathrm{is}\;\mathrm{connected}\;\mathrm{to}\;\mathrm{remote}\;\mathrm{server} \end{array}\right\}$.

Let $R\prime_{j,f}$ be the request sent by the D2DR j for file f to ID2DT i. If the file f is cached by the ID2DT i, then $X_{i,f}=1;$ otherwise $,\;X_{i,f}=0$. Similarly, if the file is cached by the BS of tier t, then $Y_{t,f}=1;$ otherwise,$\;Y_{t,f}=0$.

$S_{j}$,$\;S_{P}$, $S_{m}$, and $S_{M}$ are cache memories of the ID2DT, pico BS, micro BS, and macro BS, respectively. The overall cache hit ratio of the system is given as

(1) $$\beta =\frac{\sum_{f\in ℱ}\sum_{i\in K}\sum_{j\in ℛ}\left\{(X_{i,f}R_{j,f}\lambda_{i,j})Z_{i,j}+\left(1-Z_{i,j}\right)\left[\left(Y_{p,f}R_{j,f}\right)Z_{p,j}+\left(1-Z_{p,j}\right)\left(\left(Y_{m,f}R_{j,f}\right)Z_{m,j}+\left(1-Z_{m,j}\right)Y_{j,f}R_{j,f}Z_{M,j}\right)\right]\right\}}{\sum_{f\in ℱ}\sum_{j\in ℛ}R\prime_{j,f}}$$

One of the tasks of this research is to optimize the overall cache hit ratio of the cache enabled D2DMCN, which is expressed as follows:

(2) $$\underset{\left\{X_{i,f},Y\prime_{t,f}\right\}}{\max}\beta$$

subject to

(3) $$C_{1}:s_{D2D}<s_{p}<s_{m}<s_{M}$$

(4) $$C_{2}:x_{i,f}\in \left\{0,1\right\}$$

(5) $$C_{3}:{y\prime }_{t,f}\in \left\{0,1\right\}$$

The objective function in Equation (2) accounts for the overall cache ratio of the system. Constraint $C_{1}$ shows the limitations for the cache memory of BSs of MCN, while constraints $C_{2}$ and $C_{3}$ are binary variables. Thus, in order to maximize the overall cache hit ratio of the cache-enabled D2DMCN, the optimal variables $X_{i,f}$ and $Y_{t,f}$ are set by constructing a suitable cache placement framework to maximize the cache hit ratio. We represent the cache hit ratio as a mixed integer linear programming (MILP) problem, which is NP-hard, whose computational complexity increases exponentially with the increasing size of the D2Ds. Hence, we suggest a machine learning-based distributed solution which has less complexity and is based on prediction of popularity of newly published contents and placement of those contents which are mostly to be viewed in near future. Accordingly, the optimization objectives of this research are described below.

First, the popularity of the contents is determined, and contents are placed in ID2DTs and the MCN, with the prime goal of improving the cache hit ratio of the system using the support vector algorithm. Then, the dynamic content delivery scheme is implemented on the basis of the Hungarian algorithm, which reduces the overall time delay and power consumption. Those D2D transmitters and receivers which have a common content interest are combined in virtual clusters. An agreement is established between ID2DTs and cellular network operators. After this, each cluster is further squeezed, and those D2DRs not in the proximity of the ID2DTs are removed from the clusters. Furthermore, a content delivery policy is also necessary to minimize the delay and power consumption. The transmission rate of either ID2DTs or the MCN varies due to the difference in channel gain and channel fading. Therefore, the transmission rate $R_{i,j}$ of the cache-enabled ID2DTs can be given as

(6) $$R_{i,j}=log_{2}\left(1+\frac{P_{i,j}g_{i,j}d_{i,j}^{-\alpha }}{\sum_{i^{\prime }\neq i}P_{i\prime ,j}d_{i^{\prime },j}^{-\alpha }g_{i\prime ,j}+P_{p,j}g_{p,j}d_{p,j}^{-\alpha }+P_{m,j}g_{m,j}d_{m,j}^{-\alpha }+P_{M,j}g_{M,j}d_{M,j}^{-\alpha }+\sigma^{2}}\right)$$

where $P_{i,j}$ is the transmit power of ID2DT i to D2DR j, $g_{i,j}$ and $d_{i,j}$ are the channel gain and distance between D2D transmitter i and D2D receiver j, respectively,$\;d_{i,j}^{-\alpha }$ indicates the path loss, α shows the channel attenuation index, and $\sigma^{2}$ is the power of Gaussian noise. $P_{p,j},g_{p,j},$ and $P_{m,j},g_{m,j}$ are the transmit power and channel gains of the pico cell transmitter and micro cell transmitter, respectively, while $d_{p,j}$ and $d_{m,j}$ are the distance between the pico transmitter and D2DR j and between the micro transmitter and D2DR j, respectively. Similarly, $P_{M,j}$ is the power transmitted by the macro cell transmitter, and $g_{M,j}$ and $d_{M,j}$ are the channel gain and distance between the macro cell transmitter and D2DR j, respectively. Therefore, the delay $D_{ij}$ faced by D2DR j for content request and receiving that content from the neighboring D2D transmitter i is given as

(7) $$D_{i,j}=\frac{2d_{i,j}}{R_{i,j}}$$

Now, the power consumed to deliver content from ID2DT i to D2DR j is given as

(8) $$P_{i,j}=\frac{Pd_{i,j}\gamma_{i,j}}{g_{i,j}d_{i,j}^{-\alpha }}$$

where P is the power consumed for successful transmission of the requested content in a unit distance under ideal channel conditions, and $\gamma_{i,j}$ indicates the balance between the ideal channel and current channel. Similarly, the transmission rate $R_{p,j}$ of the pico BS is given as

(9) $$R_{p,j}=log_{2}\left(1+\frac{P_{p,j}g_{p,j}d_{p,j}^{-\alpha }}{\sum_{i}P_{i,j}g_{i,j}d_{i,j}^{-\alpha }+P_{m,j}g_{m,j}d_{m,j}^{-\alpha }+P_{M,j}g_{M,j}d_{M,j}^{-\alpha }+\sigma^{2}}\right)$$

Now, the power consumed to deliver content from pico cell transmitter p to D2DR j is given as

(10) $$P_{p,j}=\frac{Pd_{p,j}\gamma_{p,j}}{g_{p,j}d_{p,j}^{-\alpha }}$$

where $\gamma_{p,j}$ indicates the balance between the ideal channel and current channel in pico mode. The delay $D_{p,j}$ in getting the content from the neighboring pico cell transmitter p by the D2DR j is given as

(11) $$D_{p,j}=\frac{2d_{p,j}}{R_{p,j}}\;$$

Similarly, the transmission rate $R_{m,j}$ of the micro cell transmitter m can be given as

(12) $$R_{m,j}=log_{2}\left(1+\frac{P_{m,j}g_{m,j}d_{m,j}^{-\alpha }}{\underset{i}{\sum }P_{i,j}g_{i,j}d_{i,j}^{-\alpha }+P_{p,j}g_{p,j}d_{p,j}^{-\alpha }+P_{M,j}g_{M,j}d_{M,j}^{-\alpha }+\sigma^{2}}\right)$$

Now, the power consumed to deliver content from micro cell transmitter m to D2D receiver j is given as

(13) $$P_{m,j}=\frac{Pd_{m,j}\gamma_{m,j}}{g_{m,j}d_{m,j}^{-\alpha }}$$

where $\gamma_{m,j}$ shows the balance between the ideal channel and current channel in micro mode. The delay $D_{m,j}$ in getting the content from the micro cell transmitter m by the D2DR j  is given as

(14) $$D_{m,j}=\frac{2d_{m,j}}{R_{m,j}}$$

Similarly, the transmission rate $R_{M,j}$ of the macro BS M  can be given as

(15) $$R_{M,j}=log_{2}\left(1+\frac{P_{M,j}g_{M,j}d_{M,j}^{-\alpha }}{\underset{i}{\sum }P_{i,j}g_{i,j}d_{i,j}^{-\alpha }+P_{p,j}g_{p,j}d_{p,j}^{-\alpha }+P_{m,j}g_{m,j}d_{m,j}^{-\alpha }+\sigma^{2}}\right)$$

Now, the power consumed to deliver content from macro cell transmitter M to D2D receiver j is given as

(16) $$P_{M,j}=\frac{Pd_{M,j}}{g_{M,j}d_{M,j}^{-\alpha \;}}$$

The delay $\;D_{M,j}$ in getting the content f from the macro BS M by the D2DR j is given as

(17) $$D_{M,j}={\frac{2d_{M,j}}{R_{M,j}}}_{}^{}$$

Therefore, the overall delay of the system is represented by τ and is given as

(18) $$\tau =\sum_{i\in K}\sum_{j\in ℛ}\frac{2d_{i,j}}{R_{i,j}}+\sum_{j\in ℛ}\left(\frac{2d_{p,j}}{R_{p,j}}+\frac{2d_{m,j}}{R_{m,j}}+\frac{2d_{M,j}}{R_{M,j}}\right)$$

The overall power consumed by the system is

(19) $$\rho =\sum_{i\in Κ}\sum_{j\epsilon ℛ}P_{i,j}+\sum_{j\epsilon ℛ}\left(P_{p,j}+P_{m,j}+P_{M,j}\right)$$

In order to minimize the delay and power consumption of the system, we can write the relation as

(20) γ=ξτ+ϱρ

where ξ and ϱ are weight coefficients. Both τ and ρ are expected to be very small. For optimal transmission, weights need to be adjusted in such a way that the delay and the transmission power reach to an optimal balance. However, this is more challenging in the cache-enabled D2DMCN as the request sent by a user may be satisfied by multiple transmitters. These transmitters may be ID2DTs or BSs of the MCN. This means that D2DR can get its favored contents from any of the transmitters, i.e., (i) ID2DTs, (ii) pico BSs, (iii) micro BSs, or (iv) macro BSs. The selection is based on three factors: the power transmitted, the gain of the channel, and the distance between transmitter and receiver. To simplify the process, we consider only those D2DRs which are in the range of multiple ID2DTs and the MCN, and which are considered as common receivers. For this, the subscript j is replaced with j*. Hence, ID2DT is generally expressed with $i^{*}$. Thus, Equations (18)–(20) are generalized and are given as

(21) $$\tau^{\prime }=\sum_{i*}\sum_{j*}\frac{{2d}_{i*,j*}}{R_{i*j*}}$$

(22) $$\rho^{\prime }=\sum_{i*}\sum_{j*}P_{i*,j*}$$

(23) $$\gamma^{\prime }=\xi \tau^{\prime }+\varrho \rho^{\prime }$$

Now, for the selection of transmitters, we introduce a decision variable $y_{i*,j*}$. Our prime goal is to minimize the power consumption and content delay in the cache-enabled D2DMCN. For this purpose, we introduce a system reward function $R_{v}$. This system reward minimizes the total delay and power consumption of the system. The reward of the system is defined as

(24) $$R_{v}=\left(\xi \frac{{-d}_{i*,j*}}{log_{2}\left(1+\frac{P_{i*,j*}g_{i*,j*}d_{i*,j*}^{-\alpha }}{\sum_{i\prime *\neq i*}p_{i\prime *,j*}{g_{i\prime *.j*}d}_{i\prime *,j*}^{-\alpha }+\sigma^{2}}\right)}-\varrho \frac{d_{i*,j*}P}{{g_{i\prime *.j*}d}_{i\prime *,j*}^{-\alpha }}\right)$$

where ξ and ϱ are the weight coefficients indicating the significance of content delay and power consumption, respectively. These weight coefficients can be used to balance the relative importance of the content delay and power consumption. If the value of ϱ is high, then, in that the scenario, the importance of content delivery will be high. Hence, the second optimization problem is given below.

(25) $$\underset{\left\{y_{i*,j*}\right\}}{\max}\sum_{i*}\sum_{j*}{y_{i*,j*}R}_{v}$$

subject to

(26) $$C_{4}:\;y_{i*,j*}\in \{0,1\}$$

(27) $$C_{5}:\sum_{i*}y_{i*,j*}\leq 1\;,\;\forall j*$$

(28) $$C_{6}:\sum_{i*}y_{i*,j*}P_{i*,j*}\leq P_{k}^{\max},\forall i*$$

The objective function in Equation (25) accounts for the reward of the system, which is maximized when the content delay and power consumption are minimized. Constraint $C_{4}$ is a binary variable whose value is 1 when content is delivered from transmitter i* to D2DR j* and 0 otherwise. $C_{5}$ represents that D2DR j* can be served by a single transmitter i* at a time, while constraint $C_{6}$ indicates the power range of the system.

## 4. Clustering and Cache Making Strategy

K-means clustering and the support vector algorithm were used for clustering and content caching, respectively.

### 4.1. K-means Clustering and the Concept of Squeezed Cluster

The caching process becomes easier and more efficient if similar D2Ds are placed in virtual groups called clusters. These clusters are formed on the basis of the similarity of the contents, as requested by the users. There are many unsupervised learning algorithms for cluster formation, but the K-means clustering algorithm is more viable. The inputs to the algorithm are the ratings given by the users to different contents. ID2DTs are selected, and an agreement is made between ID2DTs and cellular operators. According to that agreement, the cellular operator can use the limited memory and battery power of ID2DTs; in response, the cellular operator allocates extra MBs for this purpose. After this, squeezed clusters are formed, and those D2DRs within the threshold distance range of ID2DTs are left in the cluster. This means that every ID2DT has its own squeezed cluster, and there is a fairly greater chance of D2DRs getting their requested contents from ID2DTs. Sometime, a D2DR can be in the range of two or more ID2DTs. In such a scenario, the Hungarian algorithm is used for content delivery, as discussed later.

### 4.2. Support Vector Algorithm for Estimating the Popularity Matrix and Proactive Caching

In cache-enabled D2DMCN, content caching has a direct impact on the cache hit ratio and satisfaction rate. Thus, content caching plays a pivotal role. Each user can easily find their favored contents in their neighborhood if content caching is properly carried out. The neighborhood may have a cache-enabled ID2DT or the BS of a cache-enabled MCN. This can hugely reduce the backhaul link load and transmission delays. On the other hand, if content caching is not properly designed, then the user’s frustration level will increase as they cannot find their requested content with ease. Thus, for more effective content caching, the user content request distribution plays a vital role. This will ease the process of what to cache and where to cache. Different machine learning algorithms are employed for predicting the content popularity. In our research work, we used the support vector algorithm. The attributes of the contents are numbers of likes, numbers of shares, numbers of dislikes, numbers of comments, first-day view counts, etc. First, the machine is trained, and the popularity is predicted. Then, highly popular contents are cached in the ID2DTs and BSs of-cache enabled MCNs. Few contents are cached in the limited memory of the ID2DT. MCNs are also limited in terms of cache memory. Therefore, the first problem, which is related to the overall cache hit ratio, can be solved by the support vector algorithm. The support vector machine uses the radial basis function (RBF) as a kernel for transformation. The RBF can better map feature vectors into a nonlinear space. This nonlinear property of RBF kernel transformation performs better prediction of the content popularity because it does not rely only on the linear relation between first-day view count and the view count on later days. The SVM finds the evaluation pattern in the dataset and predicts the content’s first-day view count accordingly. According to the SVM-based model, the numbers of views of contents *f* at $\theta_{t}$ can be defined as

(29) $$\overset{\wedge }{\rho }\left(f,\theta_{t},\theta_{r}\right)=\overset{S}{\underset{s=1}{\sum }}\gamma_{s.}\varphi (W(f,\theta_{r}),W\left(s,\theta_{r}\right))+G$$

where $\overset{\wedge }{\rho }\left(f,\theta_{t},\theta_{r}\right)$ indicates the predicted numbers of views for content *f* at time $\;\theta_{r}$ when a prediction is made at time $\theta_{t}$. Similarly, $\varphi \left(w,y\right)=\exp\left(\frac{{\left\|w-y\right\|}^{2}}{2\delta^{2}}\right)$ is a Gaussian RBF with δ parameter, $W\left(s,\theta_{r}\right)$ is a feature vector for content *f* at time $\theta_{r}$, $\left\{W\left(s,\theta_{r}\right)\underset{s=1}{S}\right\}$ is the support vector returned by the SVR algorithm with a set coefficient ${\left\{\gamma_{s}\right\}}_{s=1}^{S}$, and G is the intercept.

### 4.3. Clustered-SVM

**Figure 3:**
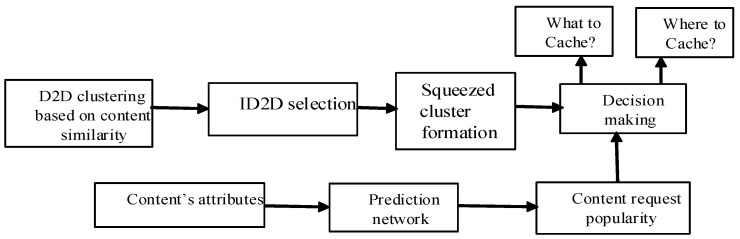
Content popularity prediction-based caching framework in D2D networks.

We combined K-means clustering and SVM, which we named clustered-SVM. As shown in Figure 3, the content popularity was predicted by SVM, and D2D clustering was carried out by K-means clustering. ID2DTs were selected and squeezed clusters were formed, consisting of those D2DRs in the proximity of ID2DTs. Then, we connected selected ID2DTs with the content popularity and decided on what to cache and where to cache. Similarly, the popularity of contents in each tier of the MCN was predicted by the SVM, and contents were cached accordingly. The two cache decision variables $X_{i,f},Y_{t,f}$ could be obtained using clustered-SVM, as given in Algorithm 1. Let $D_{1}^{\prime }$, $D^{\prime },D^{train}$ be the datasets for the distance between the D2Ds, ratings of the past requested contents, and attributes of contents, respectively, while $D^{testd2d},D^{test1},D^{test2},D^{test3}$ are the testing data of content popularity for the D2D, pico BS, micro BS, and macro BS respectively. Similarly, $D^{\prime \prime }$ is the dataset for fresh content file f request $R\prime_{j,f}$ of D2DR j. Initially, using dataset $D^{\prime },$ clusters $C_{1},\;C_{2},\ldots ,C_{c},$ having similar D2Ds, are formed using K-means clustering. Then, ID2DTs are selected and, using $D_{1}^{\prime }$, squeezed clusters $sc_{i}$ are formed, thereby removing those D2DRs not in the proximity $d_{max}$ of the selected ID2DTs. SVM is trained with $D^{train}$ and tested with $D^{testd2d}$, and the popularity of the contents is determined by predicting the first-day view count of the contents. Then, the popularity is sorted in descending order, and the most popular contents are cached in the ID2DTs in descending order until the cache memory is not fully occupied. The same process applied for the pico BS, micro BS, and macro BS. The process is repeated until all cache memories of the BSs of MCN are occupied by the popular contents. Finally, cache decision variables $X_{i,f},Y_{t,f}$ are obtained.
**Algorithm 1.** Clustered-SVM to Predict Content Popularity and Implement Decision Making for Proactive Caching in Cache-Enabled D2DMCN**Require:**$\;D^{\prime },\;D_{1}^{\prime },D^{\prime \prime },D^{train},D^{testd2d},D^{test1},D^{test2},D^{test3},S,\rho_{min}$**Ensure:**$X_{i,f},Y_{t,f}^{\prime },\;\beta$**Step 0: Clustering and contents popularity predication**1.    Load $D^{\prime }$, $D_{1}^{\prime },D^{\prime \prime },D^{train},D^{testd2d},D^{test1},\;D^{test2},D^{test3}$
2.    Create *C* clusters $C_{1},\;C_{2},\ldots \ldots ..,C_{c}$ based on data set $D^{\prime }$
3.    **for**
c=1:C
**do**4.    Randomly select a set of incentivized D2D transmitters 5.        **for**
i=1:K
**do**6.               determine vicinity of D2D transmitter, $Rmax={\left(P_{i}/\rho {\;}_{jmin}\right)}^{1/\eta_{d}}$
7.            **for**
j=1:R
**do**8.                    **if**
$d_{i,j}\;\leq \;$ Rmax9.                    $sc_{i}\leftarrow j$ // $sc_{i}\;\;is\;squeezed\;cluster\;i$
10.                   Update the D2D receiver in the squeezed cluster 11.                  
**end if**
12.           
**end for**
13.       
**end for**
14.   
**end for**
15.    Train SVM using the data set $D^{train}$
16.    Test $\;D^{testd2d},D^{test1},\;D^{test2},D^{test3}$ and predict 1st day count in $D^{testd2d},D^{test1},\;D^{test2},D^{test3}$
17.    Determine the content popularity in each case.18.    **Sort** the content popularity in descending order in each case **Step1: Content caching at incentivized D2D transmitter**19.    **for**
i=1:K do20.    Initialize the cache size $s_{i}$ and caching matrix $X_{i}$
21.        **for**
f=1:F
**do**22.          **if**
QfD2D+si^≤si//$Q_{f}^{D2D}$ is the size of the file to be cached at D2DT 23.           
$X_{i}\leftarrow 1$
24.           
si ^ ←si ^ +QfD2D
25.           
**Else**
26.            Break 27.         
**end if**
28.            Return $X_{i,f}$
29.       
**end for**
30.   
**End for**
**Step 2: Content caching at pico BS**31.    Repeat 19–30 and replacing subscripts i by p and return $Y_{p,f}$
**Step 3: Content caching at micro BS**32.    Repeat 19–30 and replacing subscripts i by m and return $Y_{m,f}$
**Step 4: Content caching at macro BS**33.    Repeat steps 19–30, and replacing subscripts i by M and return $Y_{M,f}$
34.   
**Return**
$\;\;Y_{t,f}$
**Step 5: Mode selection**35.    **for**
i=1:K
**do**36.      **for**
j=1:R do37.         **if**  $\;{\left(r_{i,j}\right)}^{-\eta_{d}}\;\geq \;{\left(r_{p,j}\right)}^{-\eta_{p}}$ **then** select D2D mode and $Z_{i,j}\leftarrow 1$
38.        **Else if** ${\left(r_{P,j}\right)}^{-\eta_{p}}\;\geq \;{\left(r_{m,j}\right)}^{-\eta_{m}}$, **then** select pico mode and $Z_{p,j}\leftarrow 1$
39.         **Else if**  ${\left(r_{mi}\right)}^{-\eta_{m}}\;\geq \;{\left(r_{M,i}\right)}^{-\eta_{M}}$, **then** select micro and $Z_{m,j}\leftarrow 1$40.          **Else** select macro mode and $Z_{M,j}\leftarrow 1$41.          
**end if**
42.       
**end for**
43.   
**end for**
**Step 6: For Cache hit ratio**44.    **Optimize** Equation **(1)**45.   
**Return**
 β


## 5. Hungarian Algorithm for Content Delivery

**Figure 4:**
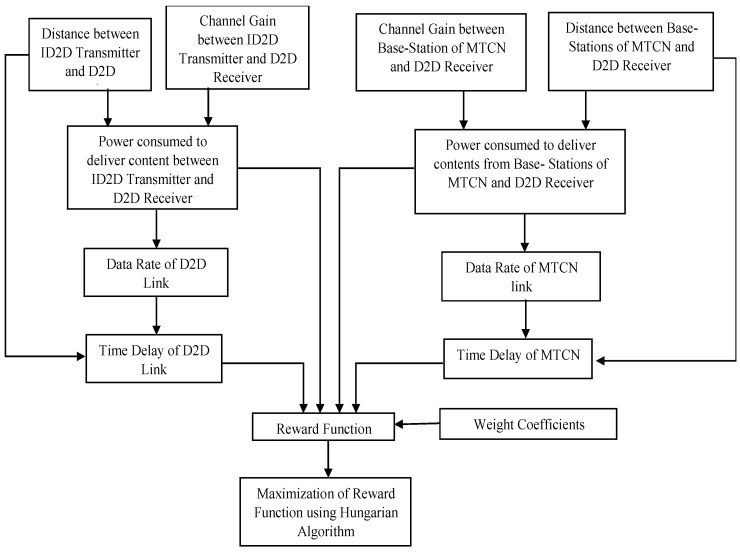
Content delivery strategy based on maximization of reward function.

The second problem in cache-enabled D2DMCN is the fast deliverance of the requested content to the D2DRs. This problem is a combinatorial assignment problem and can be solved by the Hungarian algorithm. The D2DRs can get the requested contents through D2D links or through cellular links. This content delivery becomes more cumbersome in the cache-enabled D2DMCN, since (1) the content request of a user can be satisfied by multiple transmitters at the same time, and (2) content cached by any of the transmitters may be requested by multiple receivers simultaneously. These transmitters are ID2DTs and BSs of the MCN. The said issues can be solved using the Hungarian algorithm. The Hungarian algorithm predominantly solves assignment problems. The Hungarian algorithm takes the decision of connecting D2DRs either with ID2DTs or with BSs of the MCN to get the requested contents as mentioned in the Algorithm 2 in details. To this end, the reward function is optimized. The reward function depends on the transmit power $P_{i,j}$, which is the minimum transmission power required by transmitter i and receiver j for complete transfer of information in a fading environment, the channel gain $g_{i,j},$ and the distance $d_{i,j}$ between transmitter i receiver j. Hungarian algorithm aims to maximize the reward function and minimize the power consumption and content delivery delay. The whole process is shown in Figure 4.

The D2DRs which are in the range of multiple ID2D transmitters are considered as common receivers and are designated by j*∈ℜ. Their distances and channel gains are loaded from datasets $D_{1}^{\prime }$ and $D_{2}^{\prime }$, respectively. Similarly, distance and channel gains of common D2DRs from BSs of MCN are loaded from datasets $D_{3}^{\prime }$ and $D_{4}^{\prime }$, respectively. The power consumed, transmission rate, and content delay are determined in each case, and the reward function is formed. The maximization of that reward function accounts for minimization of the overall system delay and power consumption to solve the problem of content delivery.
**Algorithm 2.** Hungarian Algorithm to Perform Content Delivery Decision Making and Optimizaion of Reward of the System1. **Definition:**2. $D_{1}^{\prime }:$ data set for distance between ID2D transmitters and D2D receiver 3. $D_{2}^{\prime }:$ data set for channel gains of link between ID2D transmitters and D2D receivers 4. $D_{3}^{\prime }:$ data set for distance between multi-tier BSs and D2D receivers 5. $D_{4}^{\prime }:$ data set for channel gains of multi-tier BSs and D2D receivers       ξ, ϱ:  Weight coefficients 6. **Require:**
$\;D_{1}^{\prime },D_{2}^{\prime },\;D_{3}^{\prime },D_{4}^{\prime },\;\xi ,\;\varrho$
7. **Ensure:**
$\;y_{i*,j*}$
8. **Load** 
$D_{1}^{\prime },D_{2}^{\prime },\;D_{3}^{\prime },\;D^{\prime \prime }$
9. **Set values of**
 ξ, ϱ
10.   **for**
t=1:3 **//**
when t=1, we have pico BS, 11.       **for**
 i=1:K **do**12.         **for**
 j=1:R
**do**
13. **Using**
$P_{i,j},\;d_{i,j},\;g_{i,j},P_{p,j},d_{p,j,\;}P_{m,j},g_{m,j},d_{m,j,\;}P_{M,j},g_{M.j}\;and\;d_{M,j}$
in(6), (9), (12) and (15) to             Compute $R_{i,j},\;\;R_{p,j},\;R_{m,j}\;and\;R_{M,j}$
14. **Using**
$R_{i,j},R_{p,j},\;R_{m,j}\;and\;R_{Mcr^{\prime }}$ in (7), (11), (13) and (16) to Compute $\;\;D_{i,j},\;D_{p,j},\;D_{m,j},D_{M,j}$
15.         **end for**
16.       **end for**
17.    **end for**
18.   **for**
 i=1:K **do**19.        **for**
j=1:R 
**do**
20.         **Find common D2DRs set among squeezed clusters and MCN**
21.       **end for**
22.    **end for**
23.   **for**
i*
**=**
$1:k^{\prime }$  do **// for general transmitters**
i* $\in K^{\prime }$24.         **for**
j* $=1:\;R^{\prime }$ do25.            **Calculate** the reward using formula (24)26.            **Use Hungarian algorithm** maximize the reward of the system27.         **end for**
28.   **end for**
29. **return** 
$y_{i*,j*}$


## 6. Performance Evaluation

### 6.1. Simulation Setup

In this section, we provide different simulation results for the performance of the D2DMCN with the proposed content caching and content delivery scheme. D2DRs can obtain contents either from ID2DTs or from multiple tiers of the cellular network. These tiers are the macrocell tier, microcell tier, and picocell tier. All tiers are in the coverage circle of macrocell with radius R = 800m. Additionally, 200 D2DRs are distributed randomly forming a Poisson distribution. The macro BS and pico BS are also installed with radii of 300 m and 150 m, respectively. Almost 10% of the D2Ds are ID2DTs, while the remainder are D2DRs. For simplicity, one ID2DT can only be connected to one D2DR at a time for file transfer. Similarly, one file can be transmitted at each time. On the other hand, the MCN has also its own receivers, which can obtain contents directly from the BSs of the MCN. Each D2D transmitter can cache up to 10 files. The size of file is similar (1 MB for simplicity). The maximum transmit power is 1 W, the sensitivity of each D2DR is $\rho_{\min}=-70\;\mathrm{dBm}$, and the path loss exponent is α=3. Different parameters and their values/types used in this research are given in Table 2.

For validation of the performance of the experiment, huge datasets were required [43,44,45]. A few datasets were selected from Movie Lens (ML) as experimental data containing records of 5000 ratings, while others were generated according to the social behavior of the users. These datasets comprised requests of the users for various contents, attributes of the contents for content popularity, distances between D2Ds located at a different locations, distances between D2Ds and multitier BSs, channel gain and transmitted power for D2D communication, channel gain and transmitted power for cellular communication, etc. For content popularity, the number of training data was 3000, and the number of testing data was 2000.

### 6.2. Numerical Results and Discussion

#### 6.2.1. Performance Evaluation of Content Caching Based on Different Learning Algorithms for D2DMCN

**Figure 5:**
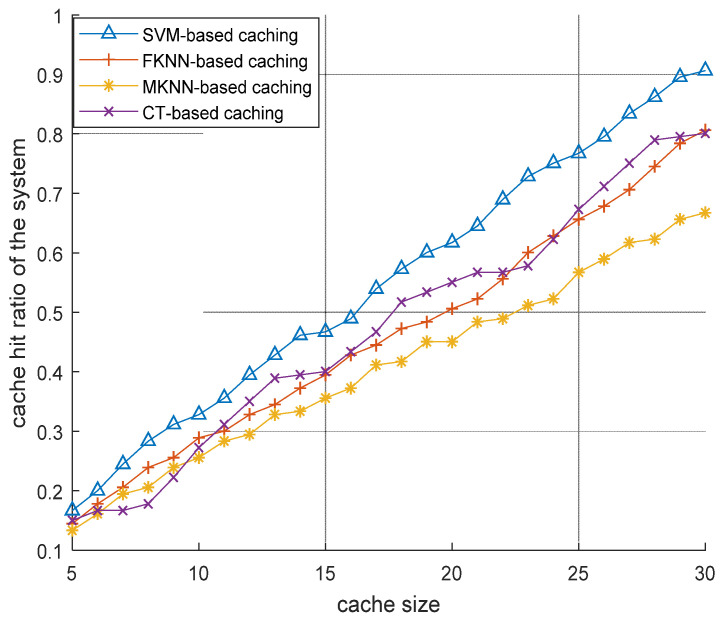
Performance evaluation of content caching based on SVM at D2D assisted multitier cellular networks.

Figure 5 depicts the overall cache hit ratio of the cache-enabled D2D assisted MTCNs. It can be observed that content caching based on SVM is better compared to the fine K-nearest neighbor (FKNN), medium K-nearest neighbor (MKNN), and complex tree algorithms. The reason is that SVM has a higher prediction accuracy owing to the fact that the RBF can better map feature vectors into a nonlinear space. This nonlinear property of RBF kernel transformation enables better prediction of the content popularity because it does not rely only on the linear relationship between first-day view count and the view count on later days. SVM finds the evaluation pattern in the dataset and predicts the content’s first-day view count accordingly.

#### 6.2.2. Performance Comparison of Different Proactive Content Caching Techniques for D2DMCN

We consider the following contents caching polices for D2DMCN:

- **Ground truth content caching method**

In this method, the most popular contents are cached in different cache-enabled transmitters without predicting the popularity of contents [42,46].

- **Random content caching method**

In this technique, random sets of content files are proactively periodically cached in different cache-enabled transmitters [47].

- **SVM-based content caching method**

The content popularity is predicated on using SVM from a training set, and the most popular contents are cached accordingly.

**Figure 6:**
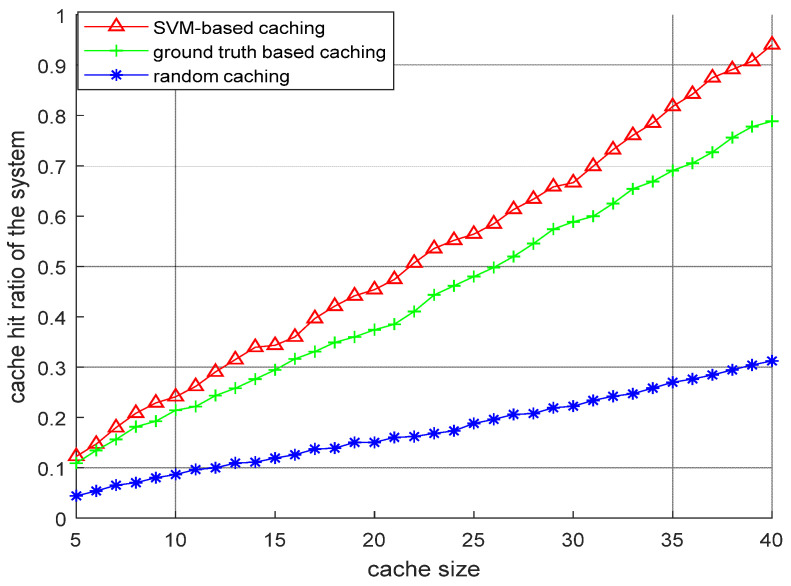
Performance comparison of different content caching techniques for D2D assisted multitier cellular networks.

Figure 6 shows the performance comparison of SVM-based caching with the ground truth caching technique and random caching technique. As expected, the SVM performed better as compared to the other two caching techniques due to the prior prediction of content popularity of newly published contents.

#### 6.2.3. Improvement in the Cache Hit Ratio Due to Inclusion of ID2DTs in MCN

**Figure 7:**
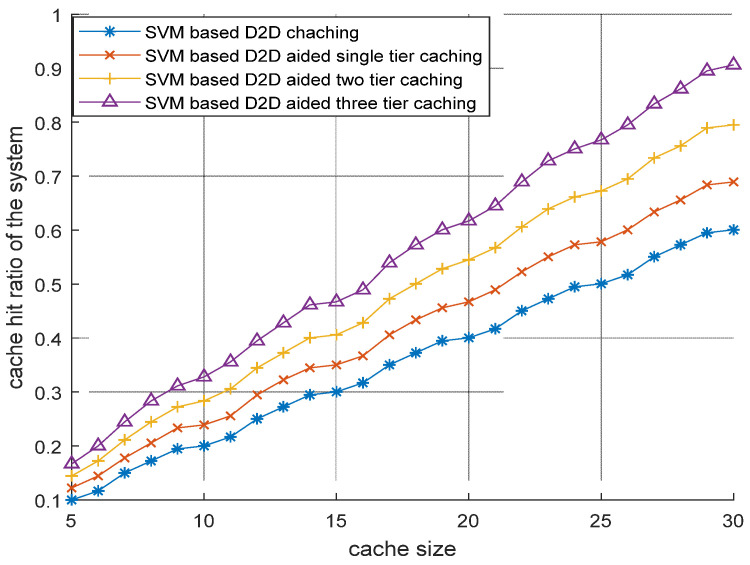
Improvement in performance due to the inclusion of D2D transmitters at various tiers of multitier cellular network.

Figure 7 describes various content caching schemes of D2DMCN. It can be observed that, if contents are cached in ID2DTs and at each tier of MCN, then D2DRs will have more chance to get their favored content either from neighboring ID2DTs or from the MCN. This means that caching at D2D is not sufficient to satisfy the varying demands of the users. This hierarchal-level caching makes the caching process more efficient.

#### 6.2.4. Content Caching at D2D Transmitters

**Figure 8:**
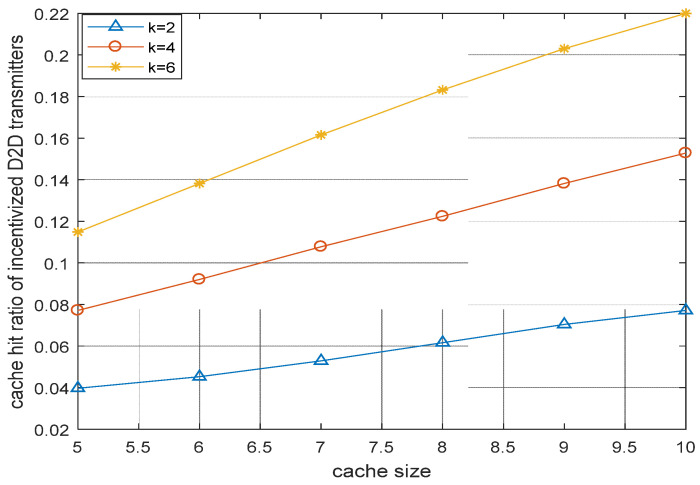
Cache hit ratio vs. cache size of incentivized D2D transmitters.

Figure 8 shows that, when the number of ID2DTs is increased, then the cache hit ratio is increased accordingly. This is due to fact that more cache-enabled ID2DTs share their cache memory for content caching.

#### 6.2.5. Impact of Varying Weight Coefficient on the Reward of the System

**Figure 9:**
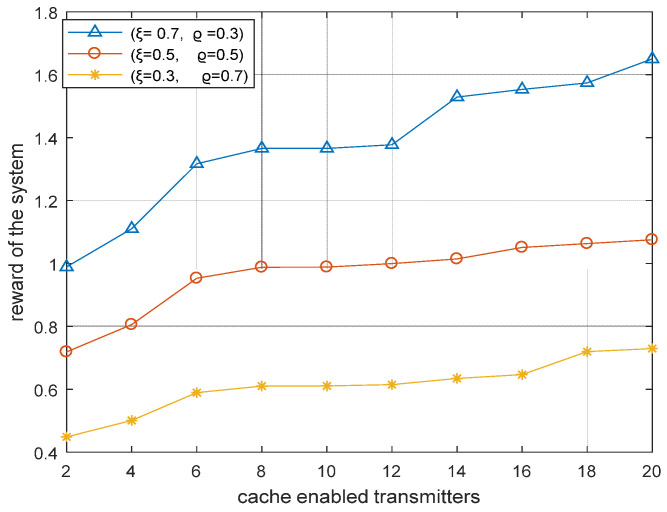
Reward of the system based on Hungarian algorithm with varying weight coefficients.

Figure 9 illustrates the reward of the system with varying values of weight coefficients ξ and ϱ. We can see that, when ξ = 0.3 and ϱ = 0.7, then the total delay of the system is low; on the other hand, when ξ = 0.7 and ϱ = 0.3, then the total power consumption of the system is low. The first case is conducive for the D2D receiver to send content requests, while the second case is conducive for the contents to be delivered from the cache-enabled transmitters. Hence, by adjusting the values of the two weight coefficients, ξ and ϱ, the performance of the system can be improved.

#### 6.2.6. Performance Comparison for the Content Delay and Power Consumption

**Figure 10:**
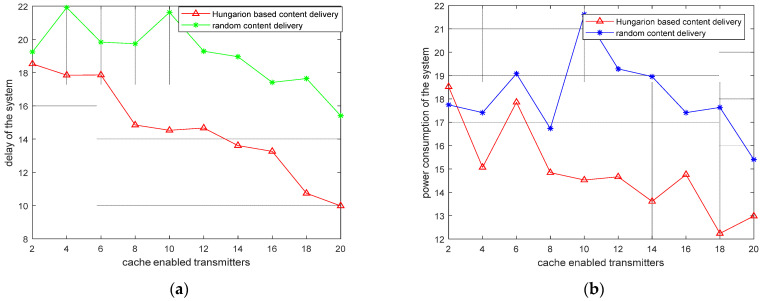
(**a**) Performance comparison of the delay of the system; (**b**) Performance comparison of the power consumption of the system.

In Figure 10a,b, we compare the Hungarian-based content delivery scheme with the random content delivery scheme [48,49,50,51,52,53] for minimizing the overall content delay and the power consumption of the system, respectively. We show that the Hungarian-based delivery scheme works much better as compared to random content delivery. Hence, the power consumption and the content delay of the system are minimized when content delivery is conducted from the D2DMCN using the Hungarian algorithm. As expected, the inclusion of cache-enabled ID2DTs in the MCN optimized the overall power consumption and delay of the system. On the other hand, if caching is performed at the tier level only, then, as expected, the power consumption and delay of the system are greater. This means that, if more ID2DTs are selected and more contents are cached in them, then the overall delay and power consumption will be decreased, and the content downloading speed will be high. Similarly, if there is no caching, then the overall delay and power consumption of the system will be much greater.

## 7. Conclusions

In this study, we proposed novel frameworks for content caching and content delivery in D2DMCN. Specifically, on the basis of the similarities of the contents, virtual clusters of D2Ds were formed. These clusters were reduced to squeeze clusters, thus leaving behind only those D2D receivers in the threshold distance range of the ID2DTs. According to the attributes of the contents, the popularity of the contents was predicted using the SVM, and popular contents were cached in the limited cache of ID2DTs and in the cache-enabled MCN. The simulation results depict that the inclusion of ID2DTs in the MCN and the use of SVM improved the overall cache hit ratio of the system. On the other hand, to solve the problem of content delivery and power consumption, a reward function of the system was constructed, which was maximized using the Hungarian algorithm. As expected, the simulation results show that the maximization of the reward function led to a minimization of the content delay and power consumption. Hence, combining the content caching and content delivery, we can greatly improve the performance of the system.

## Figures and Tables

**Table 1:** System-related parameters.

Parameters	Descriptions
$P_{i}^{max}$	maximum transmit power of ID2DT
$\rho_{min}$	minimum sensitivity of D2DR
$d_{max}={\left(P_{i}^{max}/\rho_{min}\right)}^{1/\eta_{d}}$ [42]	proximity of the ID2DT
$\eta_{d}$	path loss coefficient for D2D link
$\eta_{p}$	path loss coefficient for a link between pico BS *p* and D2DR *j*
$\eta_{m}$	path loss coefficient for a link between micro BS *m* and D2DR *j*
$\eta_{M}$	path loss coefficient for a link between macro BS *M* and D2DR *j*
$d_{p,j}$	distance between pico BS *p* and D2DR *j*
$d_{m,j}$	distance between micro BS *m* and D2DR *j*
$d_{M,j}$	distance between macro BS M and D2DR j

**Table 2:** Parameters for simulations.

Parameters	Values/Types
Total number of D2D users	200
Radius of macrocell	800 m
Radius of microcell	300 m
Radius of picocell	150 m
Total number of clusters	2
Path loss exponent	4
Thermal noise	1
Weight coefficient A and B	0 to 1
Size of each file	1
Software	MATLAB
Total number of contents	2000
System bandwidth	900 MHz
Minimum sensitivity of D2D receiver	−70 dBm
Bandwidth per channel	200 kHZ

## Data Availability

Not applicable.
